# *Rhodococcus pseudokoreensis* sp. nov. isolated from the rhizosphere of young M26 apple rootstocks

**DOI:** 10.1007/s00203-022-03079-2

**Published:** 2022-07-20

**Authors:** Peter Kämpfer, Stefanie P. Glaeser, Jochen Blom, Jacqueline Wolf, Sarah Benning, Michael Schloter, Meina Neumann-Schaal

**Affiliations:** 1 Institut für Angewandte Mikrobiologie, Justus-Liebig-Universität Giessen, 35392 Giessen, Germany; 2 Bioinformatics and Systems Biology, Giessen, 35392 Giessen, Germany; 3 Leibniz Institute DSMZ-German Collection of Microorganisms and Cell Cultures GmbH, 38124 Brunswick, Germany; 4 Research Unit for Comparative Microbiome Analysis, Helmholtz Zentrum Muenchen – National Research Center for Environmental Health, Oberschleissheim, 85758 Munich, Germany

**Keywords:** *Rhodococcus pseudokoreensis*, 16S rRNA, Genome

## Abstract

The Gram-positive strain R79^T^, isolated from the rhizosphere of young M26 apple rootstocks, was investigated by a polyphasic taxonomic approach. Phylogenetic identification based on the full-length 16S rRNA gene sequence revealed highest 16S rRNA gene sequence similarity to the type strains of *Rhodococcus wratislaviensis* (99.6%) and *Rhodococcus opacus* (99.2%) followed by *Rhodococcus imtechensis* (98.9%). All other 16S rRNA gene sequence similarities were below 98.65%. A phylogenomic tree calculated based on a whole-genome sequence also showed a distinct clustering with the type strain of *Rhodococcus koreensis.* Average nucleotide identity (ANI) values between whole-genome sequences of R79^T^ and the closest related type strains were below 95% supported the novel species status. The DNA G + C content of R79^T^ was 67.24% mol. Predominant fatty acids were C_16:0,_ C_15:0_ and C_17:1ω8c_. The strain contained MK8-H_2_ as the major respiratory quinone. The polar lipid profile consists of diphosphatidylglycerol and phosphatidylethanolamine, as well as of some unidentified lipids. The peptidoglycan type of the strain is A1γ *meso*-diaminopimelic acid. Based on the obtained genotypic and phenotypic, including chemotaxonomic data, we conclude that R79^T^ represents a novel species of the genus *Rhodococcus*, for which the name *Rhodococcus pseudokoreensis* sp. nov. is proposed. The type strain is R79^T^ (= DSM 113102^T^ = LMG 32444^T^ = CCM 9183^T^).

**Supplementary Information:**

The online version contains supplementary material available at 10.1007/s00203-022-03079-2.

## Introduction

The genus *Rhodococcus* belongs to the class of Actinobacteria and was first described by Zopf (1891). Rhodococci have been characterized as Gram-stain positive, non-motile, non-spore forming coccobacteria. The genus is highly diverse, and includes approximately 50 species which have been isolated from a large variety of habitats including soil and freshwater ecosystems (Parte 2018). In addition, rhodococci have been described as members of various holobionts (van der Geize and Dijkhuizen 2004). There are a few strains which have been described as pathogenic for humans, animals and plants, but the majority of bacteria belonging to this genus are commensals or have been described as health supporting bacteria (Bell et al. 1998; Bell et al. 1998). The high metabolic versatility may be responsible for the wide occurrence of rhodococci and due to the typically large genomes sizes.

Mainly, in terrestrial ecosystems, a number of strains has been described with the potential for bioremediation, especially for biodegradation of xenobiotics and naturally occurring organic substances that were potentially harmful for the environment (Bell et al. 1998; Larkin et al. 2006). A metagenome study by Radl et al. (2019) revealed in this respect an interesting finding: the authors could identify in soils which are affected by apple replant disease (ARD) a significantly reduced number of certain actinobacterial groups compared to non-affected control soils, revealing that it was associated with a reduced potential for degradation of phenolic compounds in the rhizosphere of apple plantlets grown in ARD-affected soils. Plant-derived phenolic compounds like phytoalexins are considered as one of the causal agents for ARD (Nicola et al. 2016; Yin et al. 2016).

In an attempt to isolate bacteria from the rhizosphere of apple plantlets, which have the potential to degrade phytoalexins in soil and can serve as a potential bioinoculum to mitigate ARD, an isolate was obtained which has been phylogenetically assigned to the genus *Rhodococcus* and named as strain R79^T^ (Benning et al. 2021). In the frame of this study, a detailed phenotypic and genotypic characterization of R79^T^ was performed. Based on morphological, physiological, biochemical, and genotypic characteristics, we propose R79^T^ as the type strain of a novel species of genus *Rhodococcus.*

## Materials and methods

### Isolation and culture condition

A bacterial strain was isolated from the rhizosphere of in vitro propagated shoots of rootstock genotype *Malus domestica* M26, which were grown in a grassland soil obtained from an experimental orchard in Ellerhoop (coordinates *x*: 53.71435; *y*: 9.770143; Schleswig–Holstein, northern Germany) in a greenhouse trial (Mahnkopp et al. 2018). After drying the rhizosphere several hours at room temperature to preselect for actinomycetes (Williams et al. 1972), 1 g of soil was mixed with 10 ml of 0.05% Tween80/50 mM TSPP (tetra sodium pyrophosphate) solution for 45 min in an overhead shaker. Serial dilutions of the soil suspension with 0.85% NaCl were spread on Actinomycete Isolation Agar (Sigma-Aldrich, Darmstadt, Germany) plates treated with 1 ml/l of 1% cycloheximide and incubated for 48 h at 28 °C. Single colonies were separated onto new plates. Long-term preservation was ensured with 25% (v/v) glycerol stocks at − 80 °C, after cultivation of isolates in Actinomyces Broth (Sigma-Aldrich) for 2 days at 28 °C. DNA from the strain was isolated, and the genome was sequenced and aligned using long read sequencing (Benning et al. 2021).

#### Molecular characterization

The four full-length 16S rRNA gene sequences derived from the genome sequence of strain R79^T^ (CP070619) were used for phylogenetic analysis. Details on genome sequencing can be found in Benning et al. (2021). The closest related type strains were determined using the EzBioCloud 16S rRNA gene identification system (Yoon et al. 2017) and by the insertion of the strain into the “All-Species Living Tree” Project database (LTP; Yarza et al. 2008) version LTP_12_2021 (Ludwig et al. 2021) using ARB version 6.0.4 (Ludwig et al. 2004). The four 16S rRNA gene sequences of strain R79^T^ were imported into the LTP database and aligned against the sequences in the database as recommended by Ludwig et al. (2021). The alignment was controlled manually before the aligned sequences of strain R79^T^ were added to the pre-existing database tree using the Quick add Parsimony method of ARB without a sequence position filter (termini option). *Rhodococcus* type strains and the type strains of related genera were included in the phylogenetic analysis. Two type strains of *Corynebacterium* species were used as outgroup. Trees were generated with different treeing methods. A maximum-likelihood tree was drawn with RAxML version 7.04 (Stamatakis 2006), GTR-GAMMA and rapid bootstrap analysis, a maximum parsimony tree with DNAPARS version 3.6 (Felsenstein 1981), and a neighbor-joining tree using ARB neighbor-joining and the Jukes–Canter model. All trees were calculated with 100 re-samplings (bootstrap analysis; Felsenstein, 1985) and based on 16S rRNA gene sequences between positions 66 to 1363 (*Escherichia coli* numbering, Brosius et al. 1978). The neighbor-joining tool was used to calculate pairwise similarity values without using an evolutionary model and EzTaxon analysis (https://www.ezbiocloud.net).

Genome sequence-based phylogenetic analyses were performed in EDGAR version 3.0 (Dieckmann et al. 2021). All genome sequences were obtained from NCBI and used to build up a private EDGAR project. The genome sequence of the type strain of *Corynebacterium diphtheriae* was used as outgroup. An amino acid sequence-based phylogenetic tree was developed based on the amino acid sequences of the core genes determined for the compared genomes. Analyses are described in detail by Dieckmann et al. (2021). Based on the clustering in the phylogenetic tree, an average nucleotide identity (ANI) matrix was generated for strain R79^T^ and the closely related type strains. The ANI matrix was based on BLASTN comparison of the genome sequences as described by Goris et al. (2007). Mean ANI values were depicted. For digital DNA–DNA hybridization (dDDH) values of the genome of closely related type strains, the Type (Strain) Genome Server (TYGS) (Meier-Kolthoff & Göker, 2019) was used.

Annotation of the genome was done using the NCBI Prokaryotic Genome Annotation Pipeline (PGAP; Li et al. 2021) and Rapid Annotation using Subsystem Technology (RAST) version 2.0 (Aziz et al. 2008) with default parameters.

For comparative genome analysis of R79^T^ and the four closest related *Rhodococcus* type strains, the genome sequences of *R. opacus* DSM 43205^T^, *R. wratislaviensis* DSM 44107^T^, *Rhodococcus jostii* DSM 44719^T^, and *R. koreensis* DSM 44498^T^ were retrieved from NCBI. All genome sequences were annotated with Prokka version 1.14.6 (Seemann 2014) with default parameters to use as input for roary version 3.13.0, a tool for rapid large-scale prokaryote pan genome analysis (Page et al. 2015), with a “minimum percentage identity for blastp” of 90 and a “percentage of isolates a gene must be in to be core” of 99. Both tools were used as implemented in the Galaxy webserver https://usegalaxy.org (Afgan et al. 2018).

#### Chemotaxonomy

For analysis of cellular fatty acids, R79^T^ and *R. koreensis* DSM 44498^T^ were grown on trypticase soy broth (TSB; BD) medium at 28 °C for 3 days. Fatty acids were analyzed as fatty acid methyl esters (FAMEs), following the protocol of Sasser (1990) for extraction, saponification, and methylation. FAMEs were separated by gas chromatography on an Agilent Technologies 6890N instrument and detected by a flame ionization detector using the Sherlock Microbial identification System (MIDI; version 6.1, TSBA40 database). Identity of fatty acids was validated by mass spectrometry (Vieira et al. 2021).

Polar lipids were extracted from freeze-dried material following the protocols described by Tindall (1990a, b), based on the method of Bligh and Dyer (1959). Separation is achieved by two-dimensional thin-layer chromatography and polar lipids were visualized using different spray reagents, specific for defined functional groups (Tindall et al. 2007).

Respiratory quinones were extracted from freeze-dried material and purified via solid-phase extraction as described previously (Vieira et al. 2021). Chromatographic separation and identification of peaks was performed by HPLC coupled to a DAD and a high-resolution mass spectrometer as described previously (Schumann et al. 2021). Isolation of peptidoglycan was performed using established protocols (Schumann 2011). Amino acids from total cell hydrolysates were analyzed via GC–MS on an Agilent Technologies 7890B GC system coupled to a 7000D GC/MS Triple Quad mass spectrometer (Schumann et al. 2021). Peptides were analyzed from partial hydrolysates by HPLC on an Agilent 1290 Infinity II LC-system coupled to an Agilent 6545 QTOF mass spectrometer, following the protocols described by Schumann (2011, 2021).

#### Phenotypic characterization

Cell morphology and motility were observed under a Zeiss light microscope at a magnification of × 1000, using cells grown for 3 days at 25 °C on trypticase soy agar (TS agar; Becton Dickinson GmbH). Gram-staining was performed by the modified Hucker method according to Gerhardt et al. (1994). Cytochrome-*c* oxidase activity was tested using Bactident oxidase test strips (Merck).

Growth of R79^T^ and *R. koreensis* DSM 44498^T^ was tested on tryptone-soy agar (TSA, Oxoid), R2A agar (R2A; Oxoid), nutrient agar (NA; BD), malt agar (Merck), glycine arginine agar (Gly/Arg; Oxoid), CASO agar (Carl Roth), K7 [0.1% (w/v) of yeast extract, peptone, and glucose, agar (15 g L^−1^), pH 6.8], M65 medium (according to DSMZ), DEV agar (DEV; Merck), Luria–Bertani (LB; Sigma-Aldrich), MacConkey agar (Oxoid), PYE [0.3% (w/v) yeast extract and 0.3% (w/v) casein peptone, agar (15 g L^−1^), pH 7.2)], nutrient broth (NU; Oxoid), marine agar (MA; Becton Dickinson), and Columbia agar with 5% sheep blood (Oxoid), respectively. All plates were incubated at 28 °C, and growth was observed after 7 days. For temperature-dependent growth, the strain was tested at 4, 10, 15, 20, 30, 36, 40, and 45 °C on TSA. NaCl tolerance was recorded at different concentrations of NaCl [0.5, 1.0, 2.0, 3.0, 4.0, 5.0, 6.0, 7.0, and 8.0 (w/v) %] in TSB and pH-dependent growth was investigated in TSB adjusted with HCl and NaOH to pH values between 4.0 and 12.0.

Additional physiological characterization was performed as described by Kämpfer et al. (1991) and Kämpfer and Kroppenstedt (1996).

Furthermore, the strain was tested with API 20 NE kit (BioMérieux) following the manufacturer’s instructions.

## Results and discussion

### Molecular and genome characteristics

**Fig. 1:**
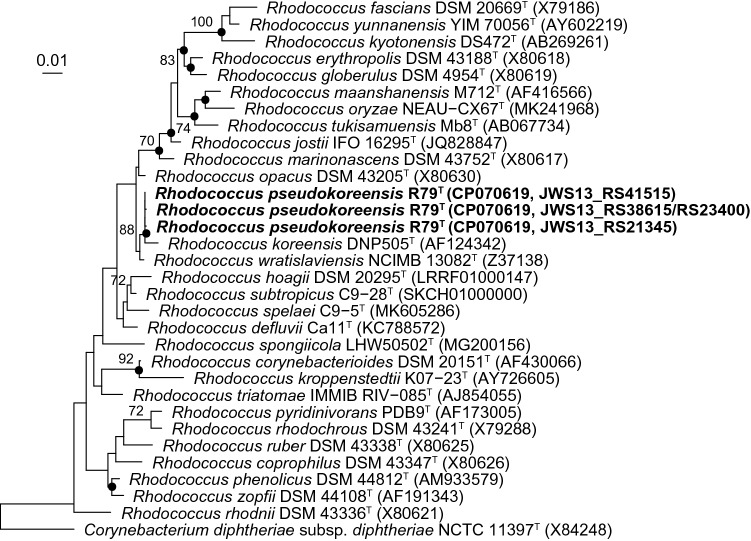
Phylogenetic placement of strain R79^T^ within the genus *Rhodococcus* based on nearly full-length 16S rRNA gene sequences. The maximum-likelihood tree was generated with the LTPs database version LTP_12_2021 using ARB based on nucleotide positions 66–1363 (according to *E. coli* numbering; Brosius et al. 1978). The respective gene sequences of two *Corynebacterium* species type strains were used as outgroup. Numbers at nodes represent bootstrap values (> 70%) based on 100 replications. Filled circles indicate nodes that were conserved in both of the trees generated with the maximum parsimony and neighbor-joining method. GenBank accession numbers are given in parentheses. Accession number of the 16S rRNA gene sequences’ first nucleotide positions is given to indicate their locations in the genome sequence of strain R79^T^. For the 16S rRNA gene sequences of strain R79^T^, the genome accession number and the locus taqs of the individual 16S rRNA gene sequences are given in brackets. Bar, 0.10 substitutions per nucleotide position

The four 16S rRNA gene sequences present in the genome of strain R79^T^ varied only at two nucleotide positions, 632 [C/T] and 1452 [T/C] (numbering according to Brosius et al. 1978). Two of the sequences were identical (see Fig. 1); others showed differences in one or both nucleotide positions. The EzTaxon analysis showed highest 16S rRNA gene sequence similarity to the type strain of *R. wratislaviensis* (99.58%), followed by *R. opacus* (99.17%), ‘*Rhodococcus imtechensis*’ (98.89%), ‘*Rhodococcus percolates*’ (98.60%), and *R. koreensis* (98.54%). The similarity values were confirmed by the calculation performed in ARB. ‘*R. percolates*’ and ‘*R. imtechensis*’ were both reclassified as heterotypic synonyms of *R. opacus* (Lee and Kim 2021) and excluded in subsequent analyses. In the phylogenetic trees drawn based on nearly full-length 16S rRNA gene sequences, strain R79^T^ clustered with high bootstrap support with the type strain of *R. koreensis* independent of the applied treeing method (Fig. 1). The above-mentioned close related type strains were placed next to the two strains.

**Fig. 2:**
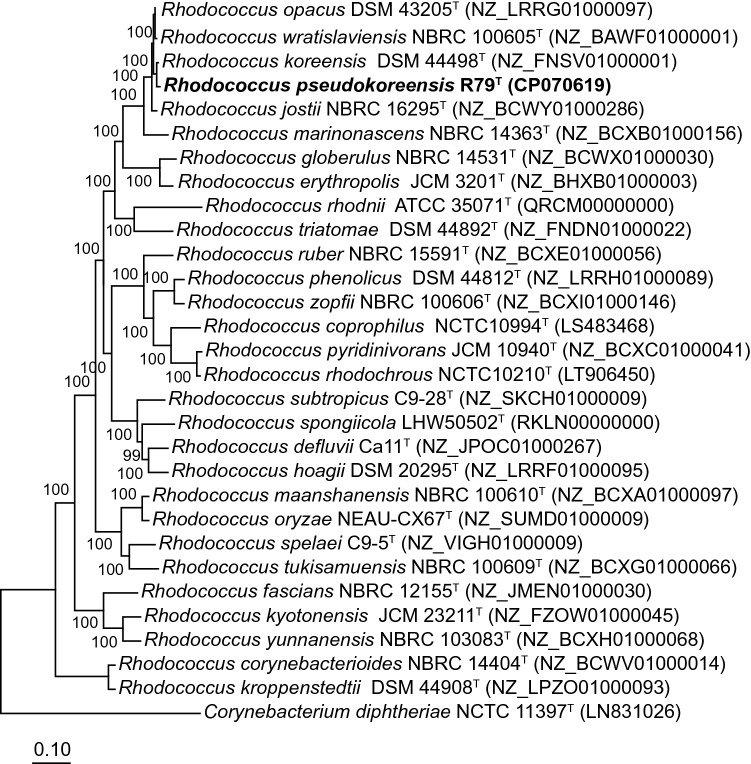
Core genome tree based on amino acid sequences of 735 shared genes. The genome sequence of *Corynebacterium diphtheriae* NCTC 11397^T^ was used as outgroup. A total of 306,769 amino acid residues were considered per genome. The tree was calculated with the fast tree method in EDGAR 3.0 (Dieckmann et al. 2021). Numbers at nodes are local support values computed by the FastTree method using the Shimodaira–Hasegawa test. Genome accession numbers are given in brackets. Bar, 0.10 substitutions per amino acid position

**Fig. 3:**
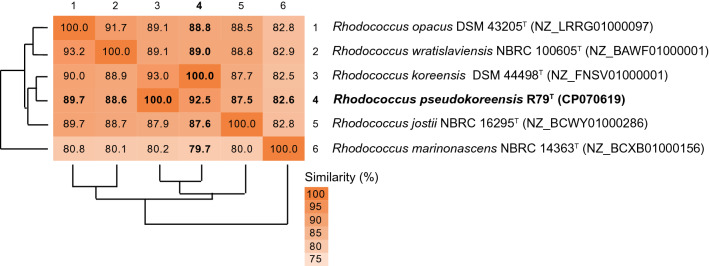
ANI value matrix comparing the genome sequences of strain R79^T^ and those of closely related type strains. Analysis was performed in EDGAR 3.0. ANI values were based on the BLASTN comparison of the genome sequences as described by Goris et al. (2016)

Phylogenetic analysis based on a set of 735 core genes (selected by the algorithms used in EDGAR for the analyzed dataset) confirmed this clustering (Fig. 2). The core gene tree was calculated with a subset of type strains of *Rhodococcus* species used for 16S rRNA gene sequence phylogeny. A total of 306,769 amino acid residues were considered per genome for the tree construction. Strain R79^T^ clustered with the type strain of *R. koreensis* in a distinct cluster with the type strains of *R. wratislaviensis*, *R. opacus*, *R. jostii*, and as outlier of the cluster *Rhodococcus marinonascens.* All those type strains were included in the comparative ANI analysis. Strain R79^T^ shared highest ANI values with the type strain of *R. koreensis* (92.5 and 93.0%) and 87.5 to 90.1% with the other type strains of *R. wratislaviensis*, *R. opacus*, and *R. jostii*, but only 79.9 and 82.6% with the type strain of *R. marinonascens* (Fig. 3)*.* The ANI analysis confirmed the close relationship to *R. koreensis* and indicates that strain R79^T^ represents a novel species, because all ANI values were below the predicted cut-off value of 95 to 96% ANI which indicates species distinction (Richter and Rosselló-Móra 2009).

The dDDH values obtained for the comparison of the genome sequences of strain R79^T^ and closely related species confirmed the ANI-based species distinction. Highest scoring values were 52.9% to *R. koreensis* NBRC 100607^T^ and 47.3% to *R*. *opacus* ATCC 51881^T^. All other dDDH values were below 45%. The predicted dDDH cut-off value for the same species is 70%.

On the linear chromosome and the 5 plasmids of R79 ^T^ (Benning et al. 2021), a total of 9243 genes were detected. As expected, many genes were related to the metabolism of secondary metabolites, including genes coding for enzymes which catalyze the degradation of aromatic compounds like biphenyl, which has a strong homology to plant-derived phytoalexins. This potential was confirmed in an in vitro assay proving the ability of the strain to degrade benzoic acid (data not shown). In addition, gene cluster for the synthesis of non-ribosomal peptides and gene cluster coding for polyketide synthesis (PKS) type I were identified, which makes the strain also of interest as a potential biocontrol agent to outcompete phytopathogens, which have been considered as part of the ARD complex (Tilston et al. 2018). Supplementary Fig. S1 shows the comparison of the genome of strain R79^T^ to genomes of the four closest related *Rhodococcus* type strains, *R. opacus*, *R. jostii*, *R. wratislaviensis*, and *R. koreensis*. 18% of all gene cluster were shared between all five individual type strains (core genes). The amount of unique gene cluster of the individual strains varied slightly between 9 and 14%, with 12% of overall gene cluster being unique to the genome of strain R79^T^. As the strain of R79^T^ was the only type strain, which was isolated from rhizosphere, we expected to detect genes related to the metabolism of plant root exudates. Indeed, genes cluster unique to the genome of R79^T^ included genes coding for enzymes catalyzing the degradation of aromatic compounds (e.g., *Biphenyl 2,3-dioxygenase subunit alpha* and *beta*, benzoate degradation genes and degradation genes for other aromatic compounds; Supplementary Table S1). Other gene cluster that was unique to R79^T^ coded for zinc transporters, enzymes catalyzing the degradation of phthalates, as well as the synthesis of antimicrobial agents (*Surfactin synthase subunit 2*, *Polyketide biosynthesis cytochrome P450 PksS*). In addition, genes driving copper resistance of the strain were detected.

### Chemotaxonomic characteristics

The fatty acid profile of strain R79^T^ contained saturated, monounsaturated, and 10-methyl-branched fatty acids, with the predominant species (> 10% of the total) being C_16:0_ (27.4%), C_15:0_ (13.5%), and C_17:1ω8c_ (13.7%) (Supplementary Table S2). Compared to the next related strain *R. koreensis*, there were minor differences in the fatty acid profile (Supplementary Table S2). In both strains, C_16:0_ was the predominant fatty acid. However, strain R79^T^ showed a higher proportion of C_15:0_ and C_16:1ω7c_ and lower levels of C_16:1ω6c_ and C_18:1ω9c_.

**Table 1:** Differentiating physiological and metabolic characteristics of R79^T^ and type strains of related species

Characteristics	1	2	3	4	5
Enzymes (API ZYM)					
Alkaline phosphatase	−	−	+	−	−
Esterase (C_4_)	−	+	+	−	−
Esterase lipase (C_8_)	+	+	+	−	−
Valine arylamidase	+	−	+	+	−
Cystine arylamidase	−	−	+	+	−
Trypsin	−	−	+	+	−
α-Chymotrypsin	−	−	+	−	−
Acid phosphatase	+	−	+	−	−
Naphthol-AS-BI-phosphohydrolase	+	+	+	+	−
α-Galactosidase	−	−	−	−	−
β-Galactosidase	+	+	−	+	+
β-Glucuronidase	−	−	−	−	−
α-Glucosidase	+	W	+	+	−
β-Glucosidase	−	−	+	+	−
Tests (API 20 NE)					
l-Tryptophane dihydrolase	−	−	−	−	−
d-Glucose fermentation	−	−	−	−	W
Arginine-dihydrolase	−	−	+	+	−
Urea production	−	−	+	+	−
Esculin hydrolysis	−	W	−	−	−
PNPG	+	+	−	+	+
Assimilation of					
d-Glucose	+	+	−	−	W
l-Arabinose	+	−	−	−	−
d-Mannose	−	−	−	−	W
d-Mannitol	+	+	−	−	−
*N*-Acetyl-d-glucosamine	+	+	−	−	W
d-Gluconate	+	+	−	−	−
Adipic acid	W	W	−	−	−
Malic acid	W	W	−	−	−

1, R79^T^; 2, *R. koreensis* DSM 44498^T^; 3, *R. wratislaviensis* DSM 44498^T^; 4. *R. opacus* DSM 43205^T^; 5, *R. jostii* DSM 44719^T^ (all data from this study); + , positive; − , negative; W, weakly positive reactions

The major isoprenoid quinone of strain R79^T^ was dehydrogenated menaquinone 8 (MK8-H_2_; 95.5%), which was also found to be the major quinone in the next related strains *R. koreensis* (Yoon et al. 2000) and *R. percolatus* (Briglia et al. 1996). Minor amounts of MK7-H_2_ (1.7%) and MK9-H_2_ (2.8%) were additionally detected.

The polar lipid profile of strain R79^T^ was consistent with those of other members of the genus *Rhodococcus*, mainly consisting of diphosphatidylglycerol and phosphatidylethanolamine (Goodfellow 1992), Furthermore, an unidentified glycophospholipid, an unidentified phospholipid and two unidentified lipids could be additionally detected. After staining with anisaldehyde, the glycophospholipid appeared as a green spot on the TLCs plate, indicating the presence of either mannose or galactose or both and might be most likely correspond to phosphatidylinositol mannoside (Supplementary Fig. S1).

The total hydrolysate of the peptidoglycan of strain R79^T^ contained the amino acids alanine, glutamate and diaminopimelic acid (DAP) in a molar ratio of 0.5 Ala:1.0 Glu:0.8 DAP. *Meso*-DAP is the only diamino acid. Hydrolysis under milder conditions showed the presence of diagnostic peptides Ala-Glu, Ala-Glu-DAP and DAP-Ala-DAP. Based on these data, the peptidoglycan type of strain R79^T^ was concluded to be A1γ (A31, *meso-*DAP-direct) which is the typical peptidoglycan type found in members of the genus *Rhodococcus* (Goodfellow, 1992).

### Physiological characteristics

The metabolic and physiological properties of strain R79^T^ different from the most closely related strains are summarized in Table 1. Additionally, cells were Gram-stain positive, oxidase-positive, non-motile and coccobacilli. The optimum growth temperature was 25–30 °C; growth occurred at 37 °C and 4 °C, but not at 45 °C. Good growth occurred at 28 °C after 72 h on TS agar, R2A, NA, malt, Gly/Arg, CASO, K7, M65, DEV, LB, PYE, NU, MA and Columbia agar. In contrast, only weak growth on MacConkey agar was observed.

## Conclusion

The reported phenotypic and genotypic characteristics congruently showed that R79^T^ represents a novel species within the genus *Rhodococcus.* The name *R. pseudokoreensis* sp. nov. is proposed, which indicates that the bacterium is closely related to *R*. *koreensis* which was described in 2000 by Yoon et al. (2000). The type strain is R79^T^.

### Description of *Rhodococcus pseudokoreensis* sp. nov.

*Rhodococcus pseudokoreensis* (pseu.do.ko.re.en´sis. Gr. masc. adj. *pseudês*, false; N.L. adj. *koreensis*, ko.re.en’sis. N.L. masc./fem. adj. *koreensis*, of or belonging to Korea, N.L. adj. *pseudokoreensis*, false *R. koreensis*).

Cells are Gram-stain positive, oxidase-positive, non-motile and coccobacilli. The optimum growth temperature is 25–30 °C; growth occurs at 37 °C and 4 °C, but not at 45 °C. NaCl concentrations are tolerated up to a concentration of 10.0% (w/v) and growth is possible at pH values from 5.5 to 10. Good growth is visible at 28 °C after 72 h on TS agar, R2A, NA, malt, Gly/Arg, CASO, K7, M65, DEV, LB, PYE, NU, MA, and Columbia agar. Weak growth on MacConkey agar was observed. Tests for indole production, fermentation of d-glucose, urease activity, hydrolysis of aesculin, and gelatin are negative.

Nitrate reduction and β-galactosidase activity are positive. Utilization of *N*-acetyl-d-glucosamine, d-fructose, d-galactose, d-gluconate, α-d-glucose, d-maltose, α-d-melibiose, (α-) l-rhamnose, d-ribose, d-sucrose, d-trehalose, i-inositol, maltitol, d-mannitol, d-sorbitol, putrescine, *cis*-aconitate, *trans*-aconitate, adipate, 4-aminobutyrate, citrate, dl-lactate, d-malate, 2-oxoglutarate, pyruvate, l-alanine, and suberate are positive. Weak assimilation of acetate, propionate, fumarate, glutarate, β-alanine, l-ornithine and 3-hydroxybenzoate, l-aspartate, l-histidine, l-phenylalanine, l-proline, 4-hydroxybenzoate, and l-leucine. No assimilation of *N*-acetyl-d-galactosamine, l-arabinose, *p*-arbutin, d-cellobiose, d-mannose, salicin, d-xylose, d-adonitol, dl-3-hydroxybutyrate, itaconate, mesaconate, l-serine, and l-tryptophan. No hydrolysis of pNP-β-d-glucuronide, pNP-α-d-glucopyranoside, pNP-β-d-glucopyranoside, pNP-phenyl-phosphonate, pNP-phosphate-disodium salt and pNP-β-d-xylopyranoside, and l-glutamate-γ-carboxy-pNA. Hydrolysis of pNP-β-d-galactopyranoside, bis-pNP-phosphate, L-proline-pNA, and pNP-phosphoryl-choline is positive.

Major fatty acids are C_16:0_, C_15:0_, and C_17:1_ɷ8c. The predominant menaquinone is MK8-H_2_. Polar lipids include diphosphatidylglycerol and phosphatidylethanolamine as diagnostic lipids. The peptidoglycan type is A1γ (A31, *meso-*DAP-direct). The genomic DNA G + C content is 67.24 mol%.

The type strain R79^T^ (= DSM 113102^T^ = LMG 32444^T^ = CCM 9183^T^) was isolated from the rhizosphere of young M26 apple plantlets grown in a grassland soil from Ellerhoop (Germany).

The GenBank/EMBL/DDBJ accession number for the complete genome sequence is CP070619 (chromosome) and CP070614 to CP070618 (plasmids).

## Supplementary Information

Below is the link to the electronic supplementary material.

Supplementary file1 (DOCX 8613 kb)

## Data Availability

The new generated sequences were uploaded to the GenBank database at the National Center for Biotechnology Information (NCBI) and are available. The complete genome sequence of strain R79^T^ has been deposited under the GenBank/EMBL/DDBJ accession numbers CP070619 (chromosome) and CP070614 to CP070618 (plasmids).

## Abbreviation

ANI: Average nucleotide identity
