# Supplementary material for: Rhodococcus pseudokoreensis sp. nov. isolated from the rhizosphere of young M26 apple rootstocks
Source: Arch Microbiol. 2022 Jul 20;204(8):505. doi: 10.1007/s00203-022-03079-2 (PMC9300504; doi:10.1007/s00203-022-03079-2)
Supplement: Supplementary file 1 — Supplementary file1 (DOCX 8613 kb) [file 203_2022_3079_MOESM1_ESM.docx]

***Supplementary material:***

***Rhodococcus pseudokoreensis* sp. nov. isolated from the rhizosphere of young M26 apple rootstocks**

Peter Kämpfer^1^, Stefanie P. Glaeser^1,^ Jochen Blom^2^, Jacqueline Wolf^3^, Sarah Benning^4^, Michael Schloter^4^, Meina Neumann-Schaal^3^

**Authors affiliation:**

^1^Institut für Angewandte Mikrobiologie, Justus-Liebig-Universität Giessen, D-35392 Giessen, Germany

^2^ Institute for Bioinformatics and Systems Biology, Giessen, D-35392 Giessen, Germany

^3^Leibniz Institute DSMZ-German Collection of Microorganisms and Cell Cultures GmbH, D-38124 Braunschweig, Germany

^4^ Research Unit for Comparative Microbiome Analysis, Helmholtz Zentrum Muenchen – National Research Center for Environmental Health, D-85758 Oberschleissheim, Germany

***Corresponding author**: Peter Kämpfer, peter.kaempfer@umwelt.uni-giessen.de

**Keywords:** *Rhodococcus pseudokoreensis*; 16S rRNA; Genome

**Abbreviation**: ANI, Average Nucleotide Identity

**Table S1** Number and annotation of gene cluster unique to *Rhodococcus pseudokoreensis* R79^T^ according to Prokka version 1.14.6 (Seemann 2014) and roary version 3.13.0 (Page et al. 2015), with a minimum percentage identity for blastp of 90, compared to the four closest related type strains of *R. opacus*, *R. jostii*, *R. koreensis* and *R. wratislaviensis*.

| Annotation of genes unique in *R. pseudokoreensis* sp. nov. R79^T^ | Number of Gene Cluster |
| --- | --- |
| (-)-trans-carveol dehydrogenase | 2 |
| (3S)-malyl-CoA thioesterase | 1 |
| (E)-2-((N-methylformamido)methylene)succinate hydrolase | 1 |
| (R)-benzylsuccinyl-CoA dehydrogenase | 4 |
| (S)-2-haloacid dehalogenase 4A | 1 |
| 1,2-epoxyphenylacetyl-CoA isomerase | 2 |
| 1,2-phenylacetyl-CoA epoxidase, subunit A | 1 |
| 1,3-propanediol dehydrogenase | 1 |
| 1,4-dihydroxy-2-naphthoyl-CoA synthase | 3 |
| 18 kDa heat shock protein | 1 |
| 1-aminocyclopropane-1-carboxylate deaminase | 1 |
| 1-deoxyxylulose-5-phosphate synthase YajO | 2 |
| 1-pyrroline-5-carboxylate dehydrogenase 2 | 1 |
| 2-(acetamidomethylene)succinate hydrolase | 2 |
| 2-(hydroxymethyl)glutarate dehydrogenase | 1 |
| 2,3,4,5-tetrahydropyridine-2,6-dicarboxylate N-acetyltransferase | 2 |
| 2,3-bisphosphoglycerate-dependent phosphoglycerate mutase | 1 |
| 2,3-dehydroadipyl-CoA hydratase | 2 |
| 2,3-dihydroxybenzoate-AMP ligase | 1 |
| 2,3-dihydroxyphenylpropionate/2,3-dihydroxicinnamic acid 1,2-dioxygenase | 1 |
| 2,3-dimethylmalate lyase | 2 |
| 2,4-dichlorophenol 6-monooxygenase | 4 |
| 2,5-dichloro-2,5-cyclohexadiene-1,4-diol dehydrogenase | 3 |
| 2,5-diketocamphane 1,2-monooxygenase 1 | 1 |
| 2-dehydro-3-deoxy-D-gluconate 5-dehydrogenase | 2 |
| 2-dehydro-3-deoxy-L-rhamnonate dehydrogenase (NAD(+)) | 1 |
| 2-haloacrylate reductase | 2 |
| 2-hydroxy-3-oxopropionate reductase | 4 |
| 2-hydroxy-6-oxo-2,4-heptadienoate hydrolase | 3 |
| 2-hydroxyhexa-2,4-dienoate hydratase | 1 |
| 2-hydroxymuconate tautomerase | 1 |
| 2-hydroxymuconic semialdehyde dehydrogenase | 1 |
| 2-hydroxypropyl-CoM lyase | 1 |
| 2-iminobutanoate/2-iminopropanoate deaminase | 1 |
| 2-keto-4-pentenoate hydratase | 1 |
| 2-methoxy-6-polyprenyl-1,4-benzoquinol methylase, mitochondrial | 1 |
| 2-methyl-1,2-propanediol dehydrogenase | 1 |
| 2-methylcitrate dehydratase | 2 |
| 2-methylcitrate dehydratase 2 | 1 |
| 2-methylcitrate synthase | 1 |
| 2-methylisocitrate lyase | 1 |
| 2-oxo-Delta(3)-4,5,5-trimethylcyclopentenylacetyl-CoA monooxygenase | 1 |
| 2-phosphosulfolactate phosphatase | 2 |
| 2-succinyl-6-hydroxy-2,4-cyclohexadiene-1-carboxylate synthase | 7 |
| 2-succinylbenzoate--CoA ligase | 7 |
| 3 beta-hydroxysteroid dehydrogenase/Delta 5-->4-isomerase | 2 |
| 3,4-dihydroxy-2-butanone 4-phosphate synthase | 1 |
| 3',5'-cyclic adenosine monophosphate phosphodiesterase CpdA | 1 |
| 3,6-diketocamphane 1,6-monooxygenase | 1 |
| 3'-5' exonuclease DinG | 1 |
| 3-alpha-hydroxycholanate dehydrogenase (NADP(+)) | 1 |
| 3-hydroxy-3-isohexenylglutaryl-CoA/hydroxy-methylglutaryl-CoA lyase | 1 |
| 3-hydroxy-3-methylglutaryl-coenzyme A reductase | 1 |
| 3-hydroxybutyryl-CoA dehydrogenase | 4 |
| 3-keto-5-aminohexanoate cleavage enzyme | 6 |
| 3-ketoacyl-CoA thiolase | 4 |
| 3-methyl-2-oxobutanoate dehydrogenase subunit alpha | 1 |
| 3-methyl-2-oxobutanoate dehydrogenase subunit beta | 1 |
| 3-methylcatechol 2,3-dioxygenase | 1 |
| 3-methylitaconate isomerase | 1 |
| 3-oxoacyl-[acyl-carrier-protein] reductase | 2 |
| 3-oxoacyl-[acyl-carrier-protein] reductase FabG | 4 |
| 3-oxoacyl-[acyl-carrier-protein] reductase FabG1 | 1 |
| 3-oxoadipyl-CoA/3-oxo-5,6-dehydrosuberyl-CoA thiolase | 2 |
| 3-oxocholest-4-en-26-oate--CoA ligase | 1 |
| 3-oxosteroid 1-dehydrogenase | 1 |
| 3-phenylpropionate/cinnamic acid dioxygenase ferredoxin subunit | 2 |
| 3-phenylpropionate-dihydrodiol/cinnamic acid-dihydrodiol dehydrogenase | 4 |
| 3-succinoylsemialdehyde-pyridine dehydrogenase | 2 |
| 3-sulfolactaldehyde dehydrogenase | 1 |
| 4-chlorobenzoyl coenzyme A dehalogenase-2 | 1 |
| 4-formylbenzenesulfonate dehydrogenase TsaC1/TsaC2 | 2 |
| 4-hydroxy-2-oxovalerate aldolase | 1 |
| 4-hydroxy-4-methyl-2-oxoglutarate aldolase/4-carboxy-4-hydroxy-2-oxoadipate aldolase | 1 |
| 4-hydroxyacetophenone monooxygenase | 1 |
| 4-hydroxymandelate oxidase | 1 |
| 4-hydroxyphenylpyruvate dioxygenase | 1 |
| 4-methyl-5-nitrocatechol 5-monooxygenase | 1 |
| 4-methylaminobutanoate oxidase (formaldehyde-forming) | 1 |
| 4-nitrophenol 2-monooxygenase, oxygenase component | 1 |
| 4-nitrophenol 4-monooxygenase/4-nitrocatechol 2-monooxygenase, reductase component | 1 |
| 5-exo-hydroxycamphor dehydrogenase | 1 |
| 5'-nucleotidase SurE | 1 |
| 6-aminohexanoate-dimer hydrolase | 1 |
| 6-deoxy-6-sulfogluconolactonase | 1 |
| 6-oxocamphor hydrolase | 1 |
| 6-phosphogluconolactonase | 1 |
| ABC-type transporter ATP-binding protein EcsA | 1 |
| Acetaldehyde dehydrogenase | 1 |
| Acetoin dehydrogenase operon transcriptional activator AcoR | 5 |
| Acetoin:2,6-dichlorophenolindophenol oxidoreductase subunit alpha | 2 |
| Acetoin:2,6-dichlorophenolindophenol oxidoreductase subunit beta | 2 |
| Acetophenone carboxylase delta subunit | 2 |
| Acetophenone carboxylase gamma subunit | 2 |
| Acetyl esterase | 4 |
| Acetyl-CoA acetyltransferase | 2 |
| Acetyl-CoA:oxalate CoA-transferase | 19 |
| Acetyl-coenzyme A synthetase | 6 |
| Aclacinomycin-N/aclacinomycin-A oxidase | 1 |
| Acryloyl-CoA reductase (NADH) | 1 |
| Acylamidase | 1 |
| Acyl-CoA dehydrogenase | 13 |
| Acyl-CoA dehydrogenase fadE12 | 7 |
| Acyl-CoA dehydrogenase FadE27 | 2 |
| Acyl-CoA dehydrogenase FadE34 | 3 |
| Acyl-CoA dehydrogenase, short-chain specific | 1 |
| Acyl-CoA thioesterase 2 | 2 |
| Adaptive-response sensory-kinase SasA | 1 |
| A-factor type gamma-butyrolactone 1'-reductase (1S-forming) | 1 |
| Alcohol dehydrogenase | 5 |
| Alcohol dehydrogenase [acceptor] | 1 |
| Alcohol dehydrogenase B | 3 |
| Aldo-keto reductase IolS | 1 |
| Aliphatic sulfonates import ATP-binding protein SsuB | 2 |
| Alkaline phosphatase D | 1 |
| Alkylmercury lyase | 1 |
| Alpha-ketoglutarate-dependent taurine dioxygenase | 3 |
| Alpha-ketoglutaric semialdehyde dehydrogenase 1 | 1 |
| Alpha-ketoglutaric semialdehyde dehydrogenase 2 | 1 |
| Altronate dehydratase | 1 |
| Anaerobic nitric oxide reductase flavorubredoxin | 1 |
| Anaerobic nitric oxide reductase transcription regulator NorR | 1 |
| Antiseptic resistance protein | 1 |
| Antitoxin | 1 |
| Antitoxin RelB | 2 |
| Antitoxin VapB2 | 1 |
| Apolipoprotein N-acyltransferase | 1 |
| Aromatic O-demethylase, cytochrome P450 subunit | 2 |
| Aromatic O-demethylase, reductase subunit | 2 |
| Arsenate reductase | 3 |
| Arsenate-mycothiol transferase ArsC1 | 3 |
| Arsenate-mycothiol transferase ArsC2 | 1 |
| Arsenical pump-driving ATPase | 2 |
| Arsenical resistance operon trans-acting repressor ArsD | 1 |
| Arsenical-resistance protein Acr3 | 2 |
| Aryl-alcohol dehydrogenase | 1 |
| Assimilatory nitrite reductase [NAD(P)H] small subunit | 1 |
| ATP-dependent RecD-like DNA helicase | 1 |
| ATP-dependent RNA helicase DeaD | 1 |
| Baeyer-Villiger monooxygenase | 3 |
| Beta-barrel assembly-enhancing protease | 1 |
| Betaine aldehyde dehydrogenase | 2 |
| Beta-ketoadipyl-CoA thiolase | 1 |
| Beta-ketothiolase BktB | 1 |
| Beta-methylmalyl-CoA dehydratase | 1 |
| Bicyclomycin resistance protein | 1 |
| Bifunctional F420 biosynthesis protein FbiB | 1 |
| Bifunctional protein FolD protein | 2 |
| Bifunctional protein PaaZ | 2 |
| Bile acid 7-alpha dehydratase | 2 |
| Bile acid-coenzyme A ligase | 1 |
| Biotin carboxyl carrier protein of acetyl-CoA carboxylase | 1 |
| Biotin carboxylase | 1 |
| Biotin/lipoyl attachment protein | 1 |
| Biphenyl 2,3-dioxygenase subunit alpha | 1 |
| Biphenyl 2,3-dioxygenase subunit beta | 1 |
| Biphenyl 2,3-dioxygenase, ferredoxin component | 1 |
| Biphenyl 2,3-dioxygenase, ferredoxin reductase component | 1 |
| Biphenyl-2,3-diol 1,2-dioxygenase | 2 |
| butyrate:acetyl-CoA coenzyme A-transferase | 1 |
| Cadmium-induced protein CadI | 1 |
| Caffeyl-CoA reductase-Etf complex subunit CarC | 3 |
| Camphor 5-monooxygenase | 2 |
| Carbamoyl-phosphate synthase large chain | 1 |
| Carbamoyl-phosphate synthase small chain | 2 |
| Carbonic anhydrase 1 | 1 |
| Carboxylesterase | 1 |
| Carboxylesterase NlhH | 1 |
| Carnitine monooxygenase oxygenase subunit | 3 |
| Carnitine monooxygenase reductase subunit | 1 |
| Carnitinyl-CoA dehydratase | 2 |
| Cell division protein FtsP | 1 |
| Chaperone protein DnaJ | 1 |
| Chaperone protein DnaK | 1 |
| Chaperone protein HscA | 1 |
| Chloroacetanilide N-alkylformylase 1, ferredoxin component | 1 |
| Chloroacetanilide N-alkylformylase 2, ferredoxin component | 1 |
| Chloronitrobenzene nitroreductase | 1 |
| Chondramide synthase cmdD | 1 |
| Chromosome partition protein Smc | 1 |
| Cis-2,3-dihydrobiphenyl-2,3-diol dehydrogenase | 1 |
| CoA-transferase/lyase DddD | 1 |
| Cobalt-precorrin-5A hydrolase | 1 |
| Coniferyl aldehyde dehydrogenase | 1 |
| Copper chaperone CopZ | 2 |
| Copper-exporting P-type ATPase | 3 |
| Copper-sensing transcriptional repressor RicR | 2 |
| Crotonobetaine/carnitine--CoA ligase | 1 |
| Crotonobetainyl-CoA reductase | 5 |
| Crotonyl-CoA carboxylase/reductase | 1 |
| Crotonyl-CoA hydratase | 3 |
| Cyanate hydratase | 1 |
| Cyclopentanol dehydrogenase | 3 |
| Cyclopentanone 1,2-monooxygenase | 1 |
| Cytochrome b6-f complex iron-sulfur subunit | 1 |
| Cytochrome c biogenesis protein Ccs1 | 1 |
| Cytochrome c biogenesis protein CcsA | 1 |
| Cytochrome P450 107B1 | 2 |
| Cytochrome P450 116 | 1 |
| Cytochrome p450 CYP199A2 | 1 |
| Cytochrome P450 monooxygenase PikC | 1 |
| Cytochrome P450-terp | 1 |
| D-alanine--D-alanyl carrier protein ligase | 2 |
| D-amino acid dehydrogenase | 1 |
| D-amino acid dehydrogenase 1 | 1 |
| D-aminopeptidase | 1 |
| D-beta-hydroxybutyrate dehydrogenase | 1 |
| Delta(1)-pyrroline-2-carboxylate reductase | 1 |
| Demethylmenaquinone methyltransferase | 1 |
| D-galactonate transporter | 2 |
| Di-/tripeptide transporter | 1 |
| Dicamba O-demethylase, ferredoxin component | 1 |
| Dihydroanticapsin 7-dehydrogenase | 5 |
| Dihydrolipoyl dehydrogenase | 1 |
| Dihydrolipoyllysine-residue acyltransferase component of branched-chain alpha-ketoacid dehydrogenase complex | 1 |
| Dihydrolipoyllysine-residue succinyltransferase component of 2-oxoglutarate dehydrogenase complex | 1 |
| Dihydropteroate synthase | 3 |
| Dimodular nonribosomal peptide synthase | 3 |
| D-inositol-3-phosphate glycosyltransferase | 1 |
| Disulfide bond formation protein D | 1 |
| Divalent metal cation transporter MntH | 1 |
| DNA ligase | 2 |
| DNA nickase | 1 |
| DNA polymerase IV | 1 |
| DNA primase | 1 |
| DNA replication and repair protein RecF | 1 |
| DNA-invertase hin | 1 |
| Drug efflux pump JefA | 1 |
| D-serine/D-alanine/glycine transporter | 1 |
| dTDP-3,4-didehydro-2,6-dideoxy-alpha-D-glucose 3-reductase | 1 |
| dTDP-glucose 4,6-dehydratase 2 | 1 |
| Dye-decolorizing peroxidase | 1 |
| Endoribonuclease MazF4 | 1 |
| Enolase | 1 |
| Enoyl-CoA-hydratase | 4 |
| Epoxyqueuosine reductase | 1 |
| ESX-1 secretion-associated protein EspI | 1 |
| Ethylmalonyl-CoA mutase | 1 |
| F420-dependent glucose-6-phosphate dehydrogenase | 4 |
| F420H(2)-dependent quinone reductase | 2 |
| Fatty acid metabolism regulator protein | 1 |
| Fatty acid oxidation complex subunit alpha | 6 |
| Fe(3+) ions import ATP-binding protein FbpC 2 | 1 |
| Fe(3+)-binding periplasmic protein | 1 |
| Ferredoxin | 1 |
| Ferredoxin-2 | 4 |
| Ferredoxin--NAD(P)(+) reductase fdr | 1 |
| Ferredoxin--NADP reductase | 2 |
| Flavin reductase | 7 |
| Flavin-dependent monooxygenase, oxygenase subunit HsaA | 2 |
| Flavin-dependent trigonelline monooxygenase, oxygenase component | 1 |
| Flavohemoprotein | 2 |
| FMN reductase (NADH) RutF | 4 |
| FMN-dependent NADH-azoreductase | 1 |
| Formyl-CoA:oxalate CoA-transferase | 5 |
| Formyltetrahydrofolate deformylase | 2 |
| Fosfomycin resistance protein AbaF | 5 |
| Fructose-1,6-bisphosphatase class 2 | 1 |
| Fumarate reductase flavoprotein subunit | 1 |
| GABA permease | 1 |
| Galactarate dehydratase (L-threo-forming) | 1 |
| Gamma-aminobutyraldehyde dehydrogenase | 1 |
| Gamma-glutamylputrescine oxidoreductase | 1 |
| Gamma-hexachlorocyclohexane dehydrochlorinase | 1 |
| Gas vesicle structural protein | 1 |
| GDP-perosamine synthase | 1 |
| Gentisate 1,2-dioxygenase | 1 |
| Gentisate transporter | 2 |
| Geranial dehydrogenase | 9 |
| Glc operon transcriptional activator | 1 |
| Gluconate 5-dehydrogenase | 2 |
| Glutamate--cysteine ligase | 1 |
| Glutarate-semialdehyde dehydrogenase | 1 |
| Glutaredoxin-like protein NrdH | 1 |
| Glutathione transport system permease protein GsiC | 2 |
| Glutathione transport system permease protein GsiD | 1 |
| Glutathione-binding protein GsiB | 2 |
| Glycerate 2-kinase | 1 |
| Glycerate dehydrogenase | 1 |
| Glyoxylate carboligase | 2 |
| Glyoxylate/hydroxypyruvate reductase A | 1 |
| Glyoxylate/hydroxypyruvate reductase B | 1 |
| Guanine deaminase | 1 |
| Haloalkane dehalogenase | 2 |
| Hca operon transcriptional activator HcaR | 3 |
| Heme A synthase | 1 |
| Hexuronate transporter | 6 |
| High-affinity branched-chain amino acid transport ATP-binding protein LivF | 1 |
| Histidine ammonia-lyase | 1 |
| HTH-type transcriptional activator CmpR | 1 |
| HTH-type transcriptional activator RhaR | 1 |
| HTH-type transcriptional activator RhaS | 3 |
| HTH-type transcriptional regulator AqdR | 1 |
| HTH-type transcriptional regulator AscG | 1 |
| HTH-type transcriptional regulator BenM | 2 |
| HTH-type transcriptional regulator BetI | 15 |
| HTH-type transcriptional regulator CdhR | 1 |
| HTH-type transcriptional regulator CmtR | 1 |
| HTH-type transcriptional regulator CynR | 6 |
| HTH-type transcriptional regulator DegA | 1 |
| HTH-type transcriptional regulator GltC | 1 |
| HTH-type transcriptional regulator HdfR | 3 |
| HTH-type transcriptional regulator HmrR | 1 |
| HTH-type transcriptional regulator KipR | 2 |
| HTH-type transcriptional regulator KmtR | 1 |
| HTH-type transcriptional regulator LutR | 2 |
| HTH-type transcriptional regulator MalT | 10 |
| HTH-type transcriptional regulator NimR | 1 |
| HTH-type transcriptional regulator PrpR | 1 |
| HTH-type transcriptional regulator TtgR | 1 |
| HTH-type transcriptional regulator VirS | 4 |
| HTH-type transcriptional regulator XynR | 2 |
| HTH-type transcriptional repressor CytR | 1 |
| HTH-type transcriptional repressor KstR2 | 2 |
| HTH-type transcriptional repressor NagR | 1 |
| HTH-type transcriptional repressor NanR | 5 |
| HTH-type transcriptional repressor NsrR | 1 |
| HTH-type transcriptional repressor RspR | 4 |
| Hydrogenase 2 maturation protease | 1 |
| Hydrolase | 1 |
| Hydroxyacylglutathione hydrolase | 3 |
| Hydroxymethylglutaryl-CoA lyase YngG | 1 |
| Hydroxypyruvate isomerase | 3 |
| Hydroxyquinol 1,2-dioxygenase | 1 |
| hypothetical protein | 1414 |
| Imidazolonepropionase | 1 |
| Inner membrane metabolite transport protein YdjE | 2 |
| Inner membrane metabolite transport protein YgcS | 3 |
| Inner membrane metabolite transport protein YhjE | 2 |
| Inosine-5'-monophosphate dehydrogenase | 2 |
| Inositol 2-dehydrogenase | 1 |
| Inositol 2-dehydrogenase/D-chiro-inositol 3-dehydrogenase | 4 |
| Inosose dehydratase | 2 |
| Insertion element IS6110 uncharacterized 12.0 kDa protein | 2 |
| Iron-dependent repressor IdeR | 1 |
| Iron-sulfur cluster carrier protein | 1 |
| IS110 family transposase ISArsp5 | 1 |
| IS110 family transposase ISBli6 | 1 |
| IS110 family transposase ISMpa1 | 1 |
| IS1380 family transposase IS1676 | 2 |
| IS1634 family transposase ISMac10 | 2 |
| IS200/IS605 family transposase ISTel3 | 5 |
| IS21 family transposase IS1415 | 1 |
| IS21 family transposase ISMt2 | 3 |
| IS256 family transposase IS1295 | 1 |
| IS256 family transposase ISArsp4 | 5 |
| IS256 family transposase ISRer3 | 1 |
| IS3 family transposase IS1141 | 1 |
| IS3 family transposase ISBli28 | 2 |
| IS3 family transposase ISBli32 | 2 |
| IS5 family transposase ISMt1 | 2 |
| IS701 family transposase ISRhosp3 | 2 |
| IS701 family transposase ISRhosp4 | 1 |
| IS701 family transposase ISSav4 | 3 |
| IS982 family transposase ISCef3 | 2 |
| Isatin hydrolase | 1 |
| ISAzo13 family transposase ISScl2 | 1 |
| ISL3 family transposase ISAar34 | 1 |
| K(+)/H(+) antiporter NhaP2 | 1 |
| Ketol-acid reductoisomerase (NADP(+)) | 1 |
| Kynurenine formamidase | 1 |
| L,D-transpeptidase 2 | 2 |
| Lactate utilization protein A | 2 |
| Lactate utilization protein B | 1 |
| Lactate utilization protein C | 1 |
| Lactose transport system permease protein LacF | 1 |
| L-amino acid dehydrogenase | 1 |
| Levodione reductase | 1 |
| L-glyceraldehyde 3-phosphate reductase | 1 |
| Limonene 1,2-monooxygenase | 3 |
| Limonene-1,2-epoxide hydrolase | 3 |
| Linear gramicidin dehydrogenase LgrE | 1 |
| Lipoprotein signal peptidase | 2 |
| Lipoyl synthase | 2 |
| L-lactate dehydrogenase | 1 |
| L-lactate transporter | 1 |
| Long-chain-fatty-acid--CoA ligase | 21 |
| Long-chain-fatty-acid--CoA ligase FadD13 | 6 |
| Long-chain-fatty-acid--CoA ligase FadD15 | 1 |
| L-threonate dehydrogenase | 1 |
| L-threonine 3-dehydrogenase | 2 |
| L-threonine dehydratase biosynthetic IlvA | 1 |
| Major myo-inositol transporter IolT | 2 |
| Malate dehydrogenase | 1 |
| Maleylacetate reductase | 1 |
| Manganese-dependent 2,3-dihydroxybiphenyl 1,2-dioxygenase | 3 |
| Medium-chain fatty-acid--CoA ligase | 1 |
| Membrane protein insertase YidC | 1 |
| Mercuric resistance operon regulatory protein | 2 |
| Metal cation efflux system protein CzcD | 1 |
| Metal-staphylopine import system permease protein CntB | 1 |
| Metapyrocatechase | 1 |
| Methanethiol S-methyltransferase 1 | 1 |
| Methyl-branched lipid omega-hydroxylase | 1 |
| Methylmalonyl-CoA carboxyltransferase 12S subunit | 1 |
| Methylthioribose kinase | 1 |
| Monoacylglycerol lipase | 1 |
| Monoterpene epsilon-lactone hydrolase | 5 |
| Multicopper oxidase MmcO | 3 |
| Multidrug resistance ABC transporter ATP-binding/permease protein BmrA | 1 |
| Multidrug resistance protein 3 | 3 |
| Multidrug resistance protein MdtH | 3 |
| Multidrug resistance protein MdtK | 1 |
| Multidrug resistance protein MdtL | 2 |
| Multifunctional non-homologous end joining protein LigD | 1 |
| Mycinamicin IV hydroxylase/epoxidase | 1 |
| Mycothiol acetyltransferase | 1 |
| Myo-inositol 2-dehydrogenase | 1 |
| Na(+)-translocating NADH-quinone reductase subunit F | 2 |
| N-acetyldiaminopimelate deacetylase | 1 |
| N-acetylglucosaminyldiphosphoundecaprenol N-acetyl-beta-D-mannosaminyltransferase | 1 |
| NAD(P) transhydrogenase subunit alpha | 1 |
| NAD(P) transhydrogenase subunit alpha part 1 | 1 |
| NAD(P) transhydrogenase subunit beta | 1 |
| NAD/NADP-dependent betaine aldehyde dehydrogenase | 2 |
| NAD-dependent methanol dehydrogenase | 2 |
| NADH dehydrogenase-like protein | 1 |
| NADH:quinone reductase | 5 |
| NADH:riboflavin 5'-phosphate oxidoreductase | 1 |
| NADH-dependent flavin reductase | 1 |
| NADP/NAD-dependent aldehyde dehydrogenase PuuC | 1 |
| NADP-dependent alcohol dehydrogenase C | 1 |
| NADP-dependent fatty aldehyde dehydrogenase | 1 |
| NADPH-dependent curcumin reductase | 1 |
| NAD-specific glutamate dehydrogenase | 2 |
| N-ethylmaleimide reductase | 1 |
| Nitrate import ATP-binding protein NrtC | 1 |
| Nitrate reductase alpha subunit | 3 |
| Nitrate reductase-like protein NarX | 3 |
| Nitric oxide synthase oxygenase | 1 |
| Nitrilase | 1 |
| Nitrilotriacetate monooxygenase component A | 1 |
| Nitroreductase NfnB | 1 |
| Novobiocin biosynthesis protein H | 1 |
| N-substituted formamide deformylase | 1 |
| N-succinylglutamate 5-semialdehyde dehydrogenase | 1 |
| Nucleoid occlusion factor SlmA | 1 |
| Nucleoid-associated protein Lsr2 | 1 |
| Outer membrane protein assembly factor BamB | 1 |
| Oxidoreductase UcpA | 2 |
| Oxygen regulatory protein NreC | 1 |
| Oxygen sensor histidine kinase response regulator DevS/DosS | 1 |
| Oxygen-dependent choline dehydrogenase | 2 |
| p-cumate 2,3-dioxygenase system, ferredoxin--NAD(+) reductase component | 1 |
| p-cumate 2,3-dioxygenase system, large oxygenase component | 1 |
| p-cumate 2,3-dioxygenase system, small oxygenase component | 1 |
| Pectin degradation repressor protein KdgR | 1 |
| Penicillin-binding protein 1F | 1 |
| Pentalenene oxygenase | 1 |
| PE-PGRS family protein PE_PGRS11 | 1 |
| Peptide deformylase | 1 |
| Peptidoglycan D,D-transpeptidase MrdA | 1 |
| Peptidoglycan deacetylase | 1 |
| Periplasmic dipeptide transport protein | 1 |
| Periplasmic nitrate reductase | 2 |
| Petrobactin import ATP-binding protein FpuD | 1 |
| Phage shock protein A | 1 |
| Phenoxybenzoate dioxygenase subunit beta | 8 |
| Phenylacetaldehyde dehydrogenase | 2 |
| Phenylacetate-coenzyme A ligase | 1 |
| Phenylacetone monooxygenase | 4 |
| Phenylalanine-specific permease | 1 |
| Phosphate-import ATP-binding protein PhnC | 1 |
| Phosphate-import permease protein PhnE | 1 |
| Phosphoadenosine phosphosulfate reductase | 2 |
| Phosphoenolpyruvate carboxylase | 1 |
| Phosphoenolpyruvate synthase | 1 |
| Phosphoenolpyruvate synthase regulatory protein | 1 |
| Phosphoglycolate phosphatase | 1 |
| Phosphoserine phosphatase 1 | 1 |
| Phthalate 4,5-dioxygenase oxygenase subunit | 1 |
| Phthalate dioxygenase reductase | 1 |
| Phthiocerol synthesis polyketide synthase type I PpsC | 2 |
| p-hydroxybenzoic acid efflux pump subunit AaeB | 1 |
| Pivalyl-CoA mutase large subunit | 1 |
| Polyketide biosynthesis cytochrome P450 PksS | 1 |
| Potassium-transporting ATPase ATP-binding subunit | 1 |
| Pristinamycin IIA synthase subunit A | 1 |
| Prodigiosin synthesizing transferase PigC | 2 |
| Proline dehydrogenase | 1 |
| Proline/betaine transporter | 11 |
| Proline-responsive transcriptional activator PutR | 1 |
| Protease HtpX | 1 |
| Protease HtpX | 1 |
| Protein adenylyltransferase SelO | 1 |
| Protein ChrB | 2 |
| Protein DipZ | 2 |
| Protein GrpE | 1 |
| Protein Ves | 1 |
| Protein YrdA | 2 |
| Protein/nucleic acid deglycase HchA | 1 |
| Pseudooxynicotine oxidase | 3 |
| Purine catabolism regulatory protein | 2 |
| putative 2,4-dienoyl-CoA reductase | 1 |
| putative 2-dehydro-3-deoxygalactonokinase DgoK1 | 1 |
| putative 3-hydroxyisobutyrate dehydrogenase | 1 |
| Putative 3-oxopropanoate dehydrogenase | 1 |
| putative 5-dehydro-4-deoxyglucarate dehydratase | 1 |
| putative 8-oxo-dGTP diphosphatase 1 | 1 |
| Putative ABC transporter arginine-binding protein 2 | 1 |
| putative ABC transporter ATP-binding protein | 2 |
| putative ABC transporter ATP-binding protein YejF | 1 |
| putative ABC transporter permease protein | 1 |
| putative ABC transporter phosphonate/phosphite binding protein PhnD2 | 1 |
| Putative acetolactate synthase large subunit IlvX | 1 |
| putative acetyl-CoA acetyltransferase | 2 |
| Putative acyl-CoA dehydrogenase FadE17 | 6 |
| putative acyl-CoA dehydrogenase fadE25 | 1 |
| Putative acyltransferase | 2 |
| putative alcohol dehydrogenase adh | 1 |
| Putative alcohol dehydrogenase D | 2 |
| Putative aldehyde dehydrogenase AldA | 2 |
| Putative alkyl/aryl-sulfatase YjcS | 2 |
| Putative aminoacrylate hydrolase RutD | 1 |
| putative BsuMI modification methylase subunit YdiO | 1 |
| putative cation efflux system protein | 1 |
| putative cation-transporting ATPase G | 2 |
| putative cystine transporter YijE | 1 |
| putative cytochrome c oxidase subunit 1 | 2 |
| putative cytochrome c oxidase subunit 3 | 1 |
| Putative cytochrome P450 126 | 1 |
| Putative cytochrome P450 YjiB | 1 |
| putative D,D-dipeptide transport system permease protein DdpC | 1 |
| putative deferrochelatase/peroxidase EfeN | 1 |
| putative D-xylose utilization operon transcriptional repressor | 2 |
| putative enoyl-CoA hydratase 1 | 3 |
| putative enoyl-CoA hydratase echA12 | 3 |
| Putative enoyl-CoA hydratase EchA13 | 2 |
| putative enoyl-CoA hydratase echA8 | 2 |
| Putative epoxidase LasC | 1 |
| putative FAD-linked oxidoreductase | 3 |
| putative ferredoxin/ferredoxin--NADP reductase | 3 |
| Putative flavin-containing monoamine oxidase AofH | 2 |
| Putative fluoride ion transporter CrcB | 2 |
| putative FMNH2-dependent monooxygenase SfnC | 1 |
| putative glycosyl hydrolase | 1 |
| putative glycosyltransferase | 1 |
| Putative glyoxylase CFP32 | 1 |
| putative GTPase | 1 |
| putative HTH-type transcriptional regulator | 1 |
| putative HTH-type transcriptional regulator RhmR | 1 |
| putative hydrogen peroxide-inducible genes activator | 1 |
| putative inactive lipase | 1 |
| Putative ketoacyl reductase | 1 |
| putative L-galactonate transporter | 1 |
| Putative metabolite transport protein YjhB | 1 |
| putative MFS-type transporter EfpA | 1 |
| putative MFS-type transporter YfcJ | 1 |
| Putative monooxygenase | 2 |
| Putative multidrug resistance protein MdtD | 3 |
| Putative mycofactocin biosynthesis transcriptional regulator MftR | 1 |
| Putative NAD(P)H nitroreductase acg | 1 |
| putative NAD-dependent oxidoreductase | 1 |
| putative NADH-specific resorcinol 4-hydroxylase | 1 |
| putative nitrate/nitrite transporter NarK2 | 1 |
| Putative non-heme bromoperoxidase BpoC | 1 |
| putative Nudix hydrolase NudL | 2 |
| putative oxidoreductase | 10 |
| putative oxidoreductase ORF5 in fasciation locus | 1 |
| Putative phenylalanine aminotransferase | 1 |
| Putative phosphoserine phosphatase 2 | 2 |
| putative protein | 13 |
| putative protein YisK | 5 |
| putative protein YyaP | 1 |
| putative quorum-quenching lactonase YtnP | 2 |
| Putative reactive intermediate deaminase TdcF | 1 |
| Putative short-chain type dehydrogenase/reductase | 3 |
| Putative short-chain type dehydrogenase/reductase/MSMEI_5872 | 4 |
| putative succinyl-CoA:3-ketoacid coenzyme A transferase subunit A | 2 |
| putative succinyl-CoA:3-ketoacid coenzyme A transferase subunit B | 2 |
| Putative tartrate transporter | 3 |
| Putative thiamine biosynthesis protein | 1 |
| Putative trans-acting enoyl reductase | 1 |
| Putative transporter YdfJ | 1 |
| Putative transporter YoaB | 2 |
| Putative transposase InsK for insertion sequence element IS150 | 1 |
| Putidaredoxin reductase CamA | 2 |
| Putrescine importer PuuP | 1 |
| Putrescine transporter PotE | 1 |
| Pyridoxal 4-dehydrogenase | 1 |
| Pyridoxal 5'-phosphate synthase subunit PdxS | 1 |
| Pyruvate dehydrogenase complex repressor | 2 |
| Pyruvate dehydrogenase E1 component subunit beta | 1 |
| Pyruvate kinase | 1 |
| Pyruvate, phosphate dikinase | 1 |
| Quinolone resistance transporter | 1 |
| Quinone oxidoreductase 1 | 5 |
| Regulatory protein AtoC | 1 |
| Release factor glutamine methyltransferase | 1 |
| Replicative DNA helicase | 1 |
| Respiratory nitrate reductase 2 beta chain | 2 |
| Response regulator protein VraR | 2 |
| Response regulator UvrY | 1 |
| Rhamnolipids biosynthesis 3-oxoacyl-[acyl-carrier-protein] reductase | 1 |
| Rhodocoxin | 2 |
| Rhodocoxin reductase | 5 |
| Riboflavin biosynthesis protein RibBA | 9 |
| Riboflavin transport system permease protein RibX | 1 |
| Ribonuclease BN | 2 |
| Ribonuclease R | 1 |
| Ribonuclease VapC2 | 1 |
| Ribonuclease VapC5 | 2 |
| Ribose import ATP-binding protein RbsA | 1 |
| Ribose import permease protein RbsC | 3 |
| Ribose operon repressor | 1 |
| RNA polymerase-associated protein RapA | 2 |
| Sarcosine oxidase subunit gamma | 1 |
| Serine protease inhibitor | 1 |
| Serine/threonine-protein kinase PknD | 9 |
| Serine/threonine-protein kinase PknK | 8 |
| Serine/threonine-protein kinase PrkC | 1 |
| Short-chain-enoyl-CoA hydratase | 10 |
| Sialic acid transporter NanT | 1 |
| Signal peptidase I W | 1 |
| Signal transduction histidine-protein kinase ArlS | 1 |
| Signal transduction histidine-protein kinase/phosphatase MprB | 1 |
| S-inosyl-L-homocysteine hydrolase | 1 |
| Solute-binding protein | 1 |
| Sorbitol dehydrogenase | 2 |
| Sporulation initiation inhibitor protein Soj | 1 |
| S-sulfocysteine synthase | 2 |
| Steroid 3-ketoacyl-CoA thiolase | 1 |
| Steroid C26-monooxygenase | 3 |
| Succinate semialdehyde dehydrogenase [NAD(P)+] Sad | 1 |
| Succinate-semialdehyde dehydrogenase | 1 |
| Succinate-semialdehyde dehydrogenase [NADP(+)] 1 | 1 |
| Succinate-semialdehyde dehydrogenase [NADP(+)] GabD | 2 |
| Succinyl-CoA--L-malate CoA-transferase beta subunit | 2 |
| Succinyl-diaminopimelate desuccinylase | 1 |
| Sulfur carrier protein FdhD | 1 |
| Surfactin synthase subunit 2 | 1 |
| Tetracenomycin-F1 monooxygenase | 1 |
| Tetracycline repressor protein class A from transposon 1721 | 1 |
| Tetracycline repressor protein class H | 1 |
| Thiamine kinase | 1 |
| Thiamine thiazole synthase | 1 |
| Thioesterase PikA5 | 1 |
| Thiol-disulfide oxidoreductase ResA | 4 |
| Toxin FitB | 1 |
| Toxin RelE | 1 |
| Trans-aconitate 2-methyltransferase | 1 |
| Transcriptional activator NphR | 5 |
| Transcriptional activator PmfR | 1 |
| Transcriptional regulator BlaI | 2 |
| Transcriptional regulator KdgR | 3 |
| Transcriptional regulator SlyA | 5 |
| Transcriptional regulator WhiB | 7 |
| Transcriptional regulator WhiB2 | 1 |
| Transcriptional regulatory protein WalR | 1 |
| Transcriptional repressor IclR | 3 |
| Transcriptional repressor Mce3R | 1 |
| Transcriptional repressor PaaX | 1 |
| Transcriptional repressor SmtB | 1 |
| Trans-feruloyl-CoA synthase FCS1 | 4 |
| tRNA 5-carboxymethoxyuridine methyltransferase | 1 |
| Tropinesterase | 1 |
| TVP38/TMEM64 family inner membrane protein YdjZ | 1 |
| Tyrocidine synthase 3 | 1 |
| Tyrosine recombinase XerC | 12 |
| Tyrosine recombinase XerD | 4 |
| Ubiquinone biosynthesis O-methyltransferase, mitochondrial | 1 |
| Universal stress protein | 1 |
| Universal stress protein/MSMEI_3859 | 1 |
| Ureidoglycolate lyase | 2 |
| Vanillate/3-O-methylgallate O-demethylase | 2 |
| Virginiamycin B lyase | 3 |
| Vitamin B12 import ATP-binding protein BtuD | 2 |
| Vitamin B12-binding protein | 1 |
| Vitamin D(3) 25-hydroxylase | 2 |
| Vitamin D3 dihydroxylase | 1 |
| Xylene monooxygenase electron transfer component | 2 |
| Xylulose-5-phosphate phosphoketolase | 1 |
| Zinc transporter ZitB | 1 |
| Zinc transporter ZupT | 1 |
| Total | 2661 |

**Table S2** Fatty acid composition of R79^T^, and type strains of related species.

The results of R79^T^ and *R. koreensis* DSM 44498^T^ were from this study. Strain: 1, R79^T^; 2, *R. koreensis* DSM 44498^T^. 3, *R. wratislaviensis*  DSM 44498^T^ (data from Kämpfer et al. 2014, obtained with the same method), 4. *R. opacus*, KACC 15303^T^ (data from Nguyen and Kim, 2016), 5, *R. jostii* CCM 4760^T^ (data from Takeuchi et al. 2002) . Values are percentage of total fatty acids. Values ≤1% in both strains are not shown. (-); ND: not determined.

| **Fatty acids** | **1** | **2** | **3** | **4** | **5** |
| --- | --- | --- | --- | --- | --- |
| C_14:0_ | 3.2 | 1.7 | 2.6 | 3.2 | 4 |
| C_15:0_ | 13.5 | 7.5 | 14.5 | ND | 8 |
| C_16:1_ ɷ7c | 7.3 | 4.4 | 12.8 | 16.2 | 13 |
| C_16:1_ ɷ6c | 4.8 | 8.0 | ND | ND | ND |
| C_16:0_ | 27.4 | 21.5 | 23.4 | 26.8 | 29 |
| C_16:0_ 10-methyl | 1.0 | 0.4 | 2.0 | ND | ND |
| C_17:1_ ɷ8c | 13.7 | 17.3 | 9.0 | 12.1 | 13 |
| C_17:0_ | 9.2 | 9.5 | 6.0 | 6.0 | 9 |
| C_17:0_ 10-methyl | 3.5 | 2.9 | 7.6 | 1.0 | ND |
| C_18:1_ ɷ9c | 6.9 | 15.5 | 5.2 | 23.8 | 16 |
| C_18:0_ | 2.0 | 2.3 | 1.2 | 3.5 | 4 |
| C_18:0_ 10-methyl | 2.6 | 4.2 | 6.5 | 2.6 | 4 |
| C_19:1_ ɷ10c | 0.8 | 1.8 | 1.4 | 1.1 | ND |

**Supplementary Figure S1:** Two dimensional thin layer chromatograms of extracted lipids of strain R79^T^. **(A)** Total lipids visualized with dodecamolybdophosphoric acid. Specific functional groups were visualized with different spraying reagents: **(B)** α-Naphthol; **(C)** Ninhydrin; **(D)** Molybdenum blue; **(E)** Anisaldehyde.

Abbreviations: DPG – Diphosphatidylglycerol; PE – Phosphatidylethanolamine; PL – unidentified phospholipid; GPL – unidentified glycophospholipid; L – unidentified lipid.


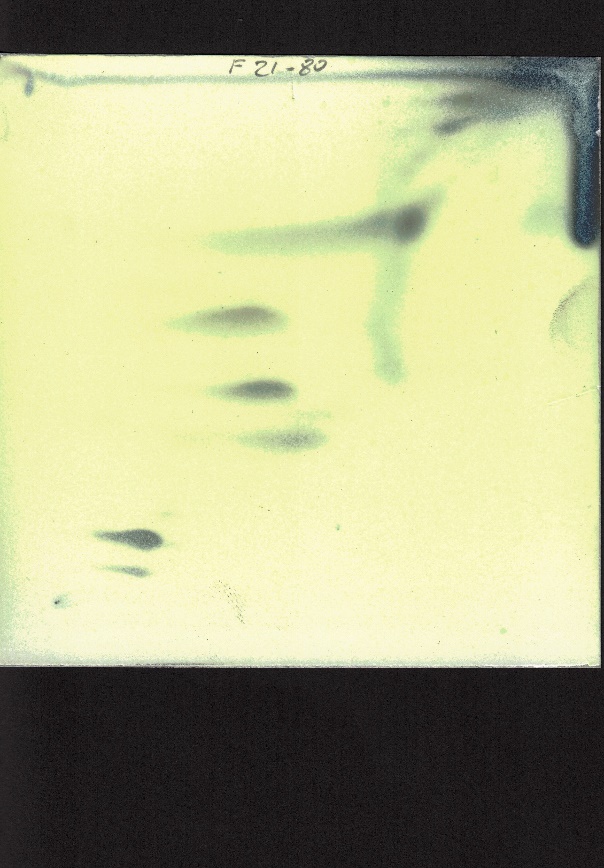


PE

A

DPG

GPL

PL

L

L


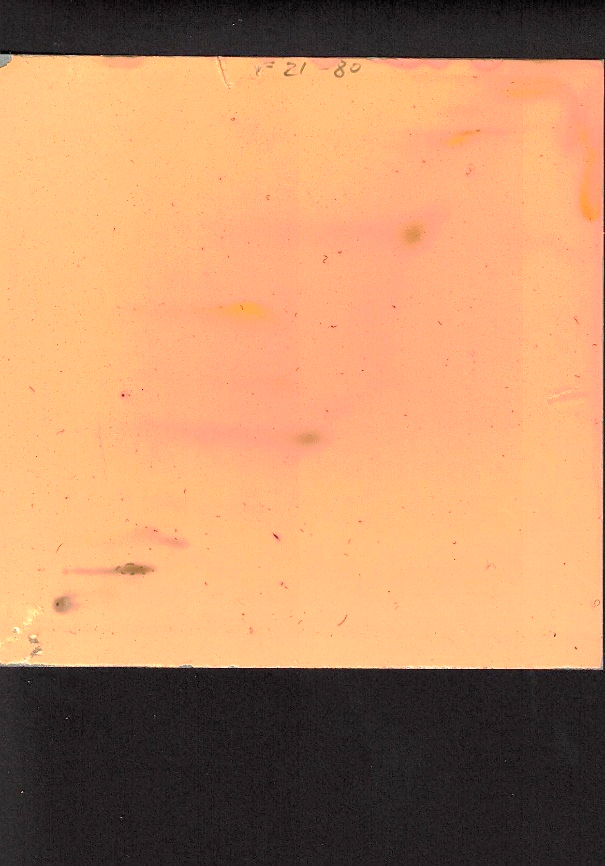


B


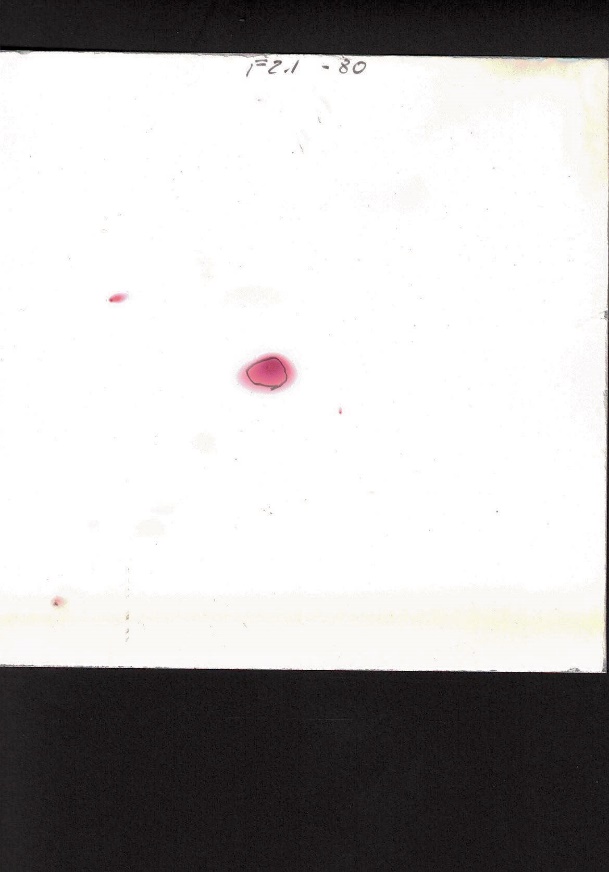


C


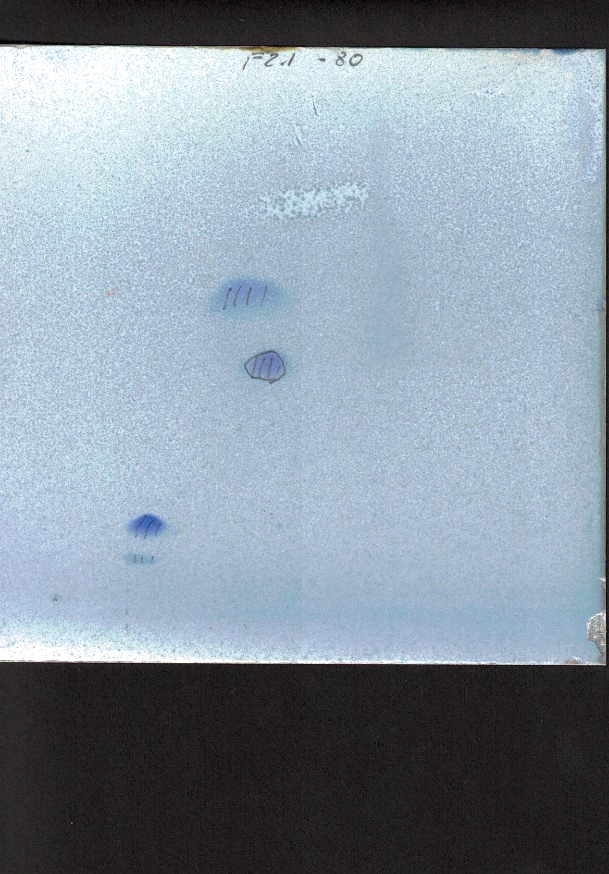


D


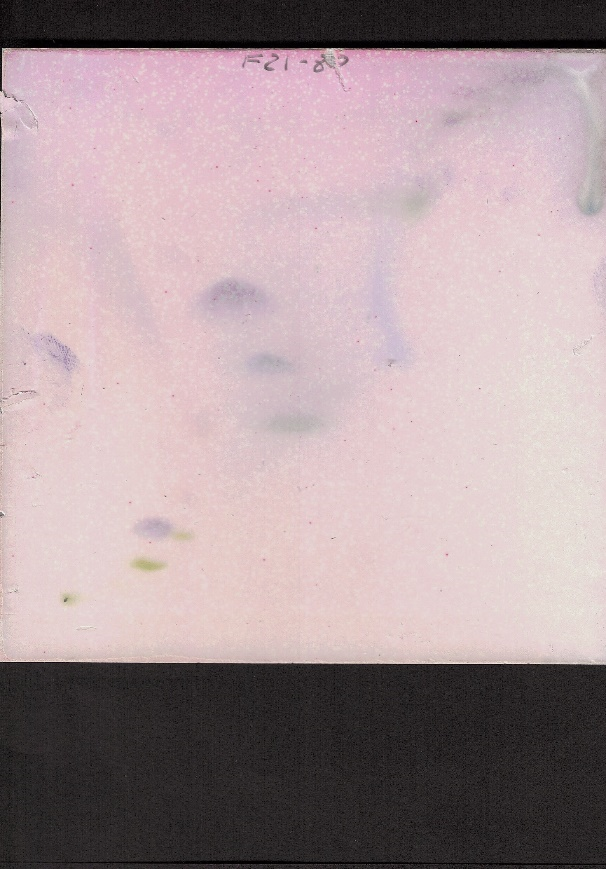


E
